# Editorial: Predicting high-risk individuals for common diseases using multi-omics and epidemiological data, volume II

**DOI:** 10.3389/fgene.2023.1280648

**Published:** 2023-09-08

**Authors:** Werner P. Veldsman, Xin Maizie Zhou, YuanWei Zhang, Bailiang Li, Lu Zhang

**Affiliations:** ^1^ Department of Computer Science, Hong Kong Baptist University, Kowloon, Hong Kong SAR, China; ^2^ Department of Biomedical Engineering, Vanderbilt University, Nashville, TN, United States; ^3^ Institute of Health and Medical Technology, Hefei Institutes of Physical Science, Chinese Academy of Sciences, Hefei, Anhui, China; ^4^ Hefei Cancer Hospital, Chinese Academy of Sciences, Hefei, Anhui, China; ^5^ Personalis, Menlo Park, CA, United States

**Keywords:** predictive genetics, disease prognosis, disease risk scoring, multi-omics, statistical modelling, machine learning

Determining predisposition to and the prognosis of diseases is often complicated by multifactorial etiology. Risk factors tend to fall along a continuum between genetic background and the environment. For some diseases, genetic and environmental risk factors interact with comparable magnitude. For others, either genetic or environmental risk factors predominate. In this Research Topic, we bring together a selection of five research articles on four important diseases with risk factors that span this continuum: systemic lupus erythematosus (SLE), head and neck squamous cell carcinoma (HNSC), hepatocellular carcinoma (HCC), and congenital heart disease (CHD). The contributing authors present high quality and creative techniques to quantify genetic risk factors using long non-coding RNAs (lncRNAs), genes associated with elevated disease susceptibility, the non-detectability of Krüpple-like factor 4 (KRF4), and nearly one million single nucleotide variants (SNVs).

Two of the five articles in the Research Topic examine the prognostic potential of lncRNAs in the context of cancer related cellular senescence. The recently discovered cuproptosis pathway (Tsvetkov et al., 2022) informed the work of Li et al. who identified ten candidate lncRNAs with previous functional annotations to the cuproptosis pathway. Using LASSO-Cox analysis, they narrowed the candidates down to five lncRNAs with prognostic value in head and neck squamous cell carcinoma. To validate their predictive model, they used a nomogram to accurately predict the survival outcome of former patients with HNSC at 1, 3 and 5 years. Li et al. furthermore found that their model is not sensitive to tumor mutation burden (TMB), which implies that their model remains applicable regardless of differences in the number of somatic mutations that there may be between sample groups. In the second paper on lncRNAs, Gao et al. also uses LASSO-Cox analysis to reduce a set of 76 candidate lncRNAs to 11 lncRNAs, this time with prognostic value in hepatocellular carcinoma. However, unlike the first paper that examines lncRNA candidates implicated in the cuproptosis pathway, Gao et al. selected candidates that all had significant oncogene-induced senescence (OIS) signatures. Using nomograms, stratified survival analysis and ROC analysis, Gao et al. demonstrates the superiority of OIS-related lncRNA subsets over four glycolysis-related and five exosome-related lncRNA subsets in predicting the survival rates of former HCC patients at 1, 3 and 5 years.

The third article addresses the problem of missed diagnoses of congenital heart disease. In this article, Tan et al. carried out a gene mining exercise on a cohort of 121 CHD patients of which they found about a third to have CHD related chromosomal abnormalities or known gene variants associated with CHD. Their discovery rate of CHD by chromosomal abnormalities was similar to that obtained by other research groups, however, Tan et al. reports that they could improve the variant based discovery rate by almost 5%, which they attribute to extensive gene set curation and in-depth manual interpretation. The primary outcome of their study is the identification of eight new CHD-related genes: *SYNE2*, *MYLK*, *PKP2*, *TRPM4*, *MIB1*, *TCAP*, *SON*, and *DSP*. In addition, they propose a set of 86 genes as candidate CHD-related genes.

In the fourth article, Chen et al. report results of a retrospective study on the topic of hepatocellular carcinoma, which is the most prevalent form of liver cancer worldwide. In contrast to the other articles, their focus was not on the presence of causative factors, but rather the absence of a known tumor suppressor by the name of Krüpple-like factor 4 (KRF4). Using a combination of bioinformatic techniques and immunohistochemistry (IHC), Chen et al. provide empirical support that the absence of KLF4 affects the tumor immune microenvironment in HCC. They report that the nomograms constructed using customized KLF4 expression scores, tumor differentiation status and TNM staging have higher prognostic accuracy than nomograms using TNM staging only. Chen et al. then experimentally validate the link between immune cell infiltration and KLF4 expression by IHC in TMA, which confirms that CD8+T and macrophage levels are significantly elevated in HCC patients with high KLF4 expression. They conclude from these results that KLF4 expression levels have prognostic value and that it may inform immunotherapy response.

The final article shifts the focus from prognosis to early detection. Here, Ma et al. uses a machine learning approach to improve on a previously reported polygenic risk score (PRS) model for the early detection of systematic lupus erythematosus. In their study, Ma et al. tested the performance of three machine learning models on a stratified Chinese/European cohort numbering nearly twenty thousand. Using the area under the receiver-operating characteristic curve (AUROC) as a performance metric, they report that random forest (RF) models, support vector machines (SVMs) and artificial neural networks (ANNs) all perform better than the PRS model, with the RF model showing a best performing 13% improvement over the PRS model for the Chinese cohort. The robustness of the Ma et al. RF model is then validated on the European cohort where it shows a 17% improvement on the PRS model, albeit with a lower sensitivity than on the Chinese cohort.

Although we do not include any articles describing studies using epidemiological data in this second volume on the Research Topic of predictive genetics, the exceptional quality of the research carried out using multi-omics data complements our first volume well. The 32 authors whose research are presented here have used genomic, transcriptomic, proteomic, and other cellular data to improve on previous disease susceptibility and prognosis scoring systems. These studies provide an informative and comprehensive snapshot of the state-of-the-art in the field of disease susceptibility and prognosis prediction.
